# Infection of the malaria vector *Anopheles coluzzii* with the entomopathogenic bacteria *Chromobacterium anophelis* sp. nov. IRSSSOUMB001 reduces larval survival and adult reproductive potential

**DOI:** 10.1186/s12936-023-04551-0

**Published:** 2023-04-13

**Authors:** Edounou Jacques Gnambani, Etienne Bilgo, Roch K. Dabiré, Adrien Marie Gaston Belem, Abdoulaye Diabaté

**Affiliations:** 1 Institut de Recherche en Sciences de la Santé (IRSS) Direction Régionale de l’Ouest (DRO)/CNRST, Bobo-Dioulasso, Burkina Faso; 2 Institut National de Santé Publique (INSP), Centre Muraz, Bobo Dioulasso, Burkina Faso; 3 Centre d’Excellence Africain en Innovations Biotechnologiques Pour l’Elimination des Maladies à Transmission Vectorielle (CEA-ITECH/MTV)/Université Nazi Boni (UNB), Bobo Dioulasso, Burkina Faso; 4 Université Nazi Boni (UNB), Bobo-Dioulasso, Burkina Faso

**Keywords:** *Chromobacterium anophelis* sp. nov IRSSSOUMB001, Mosquito larvae, Insemination rate, Wings, *Anopheles coluzzii*, Malaria

## Abstract

**Background:**

Vector control tools are urgently needed to control malaria transmission in Africa. A native strain of *Chromobacterium* sp. from Burkina Faso was recently isolated and preliminarily named *Chromobacterium anophelis* sp. nov. IRSSSOUMB001. In bioassays, this bacterium showed a promising virulence against adult mosquitoes and reduces their blood feeding propensity and fecundity. The current study assessed the entomopathogenic effects of *C. anophelis* IRSSSOUMB001 on larval stages of mosquitoes, as well as its impacts on infected mosquitoes reproductive capacity and trans-generational effects.

**Methods:**

Virulence on larvae and interference with insemination were assayed by co-incubation with *C. anophelis* IRSSSOUMB001 at a range of 10^4^ to 10^8^ cfu/ml. Trans-generational effects were determined by measuring body size differences of progeny from infected vs. uninfected parent mosquitoes using wing size as a proxy.

**Results:**

*Chromobacterium anophelis* IRSSSOUMB001 killed larvae of the pyrethroid-resistant *Anopheles coluzzii* with LT_80_ of ~ 1.75 ± 0.14 days at 10^8^ cfu/ml in larval breeding trays. Reproductive success was reduced as a measure of insemination rate from 95 ± 1.99% to 21 ± 3.76% for the infected females. There was a difference in wing sizes between control and infected mosquito offsprings from 2.55 ± 0.17 mm to 2.1 ± 0.21 mm in infected females, and from 2.43 ± 0.13 mm to 1.99 ± 0.15 mm in infected males.

**Conclusions:**

This study showed that *C. anophelis* IRSSSOUMB001 was highly virulent against larvae of insecticide-resistant *Anopheles coluzzii*, and reduced both mosquito reproduction capacity and offspring fitness. Additional laboratory, field, safety and social acceptance studies are needed to draw firm conclusions about the practical utility of this bacterial strain for malaria vector control.

**Supplementary Information:**

The online version contains supplementary material available at 10.1186/s12936-023-04551-0.

## Background

Despite the emergence of resistance and environmental concerns, chemical insecticide application remains the most common method for mosquito vector control [1]. Recent evidence suggests that progress in global malaria control has stalled, with an estimated 241 million malaria cases during 2020 among 85 malaria endemic countries, and an increase in malaria incidence in the region of Africa [2]. This stagnation and regression in disease control correlates with increasing reports of insecticide resistance, which poses a growing challenge to malaria vector control programmes. Comprehensive and integrated global, regional and national plans will need to be developed and implemented to manage insecticide resistance [1, 2]. However, as important as insecticide resistance management programme are, it is crucial to develop new vector control tools as soon as possible that will provide additional options for vector management. In the past decade, there has been intense interest in the use of biological control strategies, which aim to suppress insect vector populations by introducing endobiotic bacteria into wild populations [3–6]. A number of approaches are focused on the development of naturally-occurring or genetically engineered microorganisms as biological control agents to either block the development of the malaria parasite within the *Anopheles* vector [7–9], or to kill the vector itself [8, 10, 11]. Despite intensive efforts to develop entomopathogenic microorganisms as biocontrol agents against malaria vectors, most of these efforts have failed to meet expectations due to functional or practical limitations [12]. For example, bacteria such as *Bacillu*s *thuringiensis* var. israelensis (Bti) and *Bacillus sphaericus* (Bs) show no residual persistence post-application [13]. Interestingly, new and promising microbe-based approaches such as the use of the bacteria *Wolbachia* spp. and some eukaryotic *Microsporidia* (MB) are under investigation for malaria control [14–17]. A major caveat for translating these results from laboratory to the field involves several development steps that need to be completed before they can be used for malaria control.

Among the promising microbe-based vector control tools are bacteria in the genus *Chromobacterium*, such as *Chromobacterium vaccinii* [7] and *Chromobacterium* sp. Panama (*Csp_P*), which have insecticidal activity across different species of mosquitoes, including *Aedes aegypti* and *Anopheles gambiae *sensu stricto (*s.s.*) [8]. Additionally, Caragata et al*.*, [9] demonstrated that a non-live preparation of *Csp_P* was a highly effective larval mosquito biopesticide. Despite efforts to develop entomopathogenic *Chromobacterium* as biocontrol agents against malaria vectors, most of the strains under investigation were isolated outside of endemic regions of Africa. Our strategy has been focused on the development of Chromobacterium as a biological control agent based on the assumption that local isolates are adapted to kill local mosquitoes and have evolved to survive local conditions. (i.e. rainy season heat, sunlight and humidity).

A new strain of *Chromobacterium* sp., formerly but incorrectly identified as *Chromobacterium violaceum*, was isolated in Burkina Faso [10]. The laboratory infection of insecticide‑resistant malaria vector *Anopheles coluzzii* with this new strain of *Chromobacterium* resulted in high mortality, reduced mosquito blood feeding propensity, and almost eliminated fecundity [10]. Whole genome sequence and molecular phylogeny place this strain within the genus *Chromobacterium*, but outside any recognized species of *Chromobacterium.* For the purposes of the current study, the isolate is referred to as Chromobacterium anophelis sp. nov. strain IRSSSOUMB001. In the present study, the mosquitocidal properties of C. anophelis sp. nov. IRSSSOUMB001 against the larval stages of malaria vector Anopheles coluzzii were further explored, along with an investigation into its impact on reproductive traits within adult mosquitoes and its transgenerational impacts on mosquito fitness.

## Methods

### Mosquito colony maintenance and PCR determination of *kdr* levels

F1 progeny of *An. coluzzii* reared from larval collections at Kou Valley (11°23′ N, 4°24′ W) were used for bioassays. Mosquitoes from these areas are highly resistant to multiple insecticides currently used for malaria control [18]. First generation adult mosquitoes that had emerged from pupae were immediately sexed to prohibit any mating. Virgin males and females were kept in separate 30 × 30 × 30 cm cages. Sterilized cotton, filter paper, and nets were used to maintain the cages as aseptic as possible. Only 2–5 day-old non-blood-fed females were used for bioassays, which were carried out at 25 ± 2 °C and 80 ± 10% relative humidity., The level of *kdr* mutation within a subsample of mosquitoes (N = 291) was performed using the PCR protocol and primer sequences previously described [19]. Only the mutation L1014F was tracked because it is the most common in West Africa, whereas the L1014S mutation is confined to East Africa [19].

### Bacterial cultures and preparations for bioassays

*Chromobacterium anophelis* sp. nov. strain IRSSSOUMB001 were plated out, maintained and grown on bromocresol purple lactose agar. The protocol described in Ramirez et al. [8] was followed to grow bacteria in planktonic conditions. The estimation of the number of bacterial cells in diluted planktonic cultures was carried out using the improved Neubauer haemocytometer. In addition, the number of bacterial cells was also checked by a densitometer by measuring the optical density (OD) according to McFarland 0.5 standards. The McFarland 0.5 standard corresponds approximately to a homogeneous bacterial suspension of 1.5 × 10^8^ bacterial cells per ml.

### Bacterial infection formulation

Mosquitoes used for bioassays were maintained on 6% glucose for 2–5 days post emergence without antibiotics. Mosquitoes were then starved overnight and fed for 24 h on cotton balls moistened with a 6% glucose solution containing *C. anophelis* IRSSSOUMB001 at concentrations ranging from 10^4^ to 10^8^ cfu/ml, depending on the bioassay as previously described [10].

### Exposure of *Anopheles coluzzii* larvae to *Chromobacterium anophelis* sp. nov. IRSSSOUMB001

For this bioassay, larvae were bred in TetraMin^®^ ad libitum in individual cups. Approximately 600 L3 *An. coluzzii* larvae collected from breeding sites at Valley du Kou (11°23′ N, 4°24′ W) were mixed with C. anophelis IRSSSOUMB001 at five serial dilution concentrations from 108 to 104 cfu/ml (4 replicates of 30 mosquitoes per concentration). Control batches of L3 Larvae (4 replicates of 30 mosquitoes) were exposed to blank formulation without any *C. anophelis* IRSSSOUMB001. Dead larvae were recorded daily over 3 days before pupal stage.

### Effect of *C. anophelis* IRSSSOUMB001 on reproductive fitness and body size

The impact of *C. anophelis* IRSSSOUMB001 on mosquito reproductive fitness was measured by determining the effect of co-incubation with bacteria on insemination rates of female mosquitoes, and by measuring wing lengths as a proxy for offspring body size. 3–5 day-old virgin males and females of *An. coluzzii* were fed a 5% glucose solution containing 10^6^ CFU/ml from moistened cotton balls in a 30 × 30 × 30 cm cage for 24 h. The control group was fed sterile 5% glucose solution for 24 h without any bacteria. In order to access the impact of co-incubation on insemination rates, crosses were performed with a sex ratio of 2:1 (240 males for 120 females) (Table 1). The mosquitoes were allowed to mate for 0.5, 1, or 24 h in 30 × 30 × 30 cm cages. Following the three mating times, 120 females were withdrawn from each cage, and the next day spermathecae of female mosquitoes from each group were dissected. Their insemination status was assessed by microscopy at 400 × looking at the presence of sperms in spermathecae of female mosquitoes.

**Table 1:** Crosses between males and females based on infection status

	Infected females (IF)	non-infected females (nIF)
Infected Males (IM)	IF X IM	nIF X IM
non-Infected Males (nIM)	IF X nIM	nIF X nIM

IM infected males, *IF* infected females, *nIM*  non-infected males, *nIF* non-infected females

Inseminated female mosquitoes were exposed to *C. anophelis* IRSSSOUMB001 at 10^6^ cfu/ml for 24 h. Three hundred L3 larvae from eggs oviposited by uninfected or infected females were placed in larval bowls containing 800 ml distilled water. Larvae were also reared under standard conditions in the insectary avoid differences due to biotic and abiotic fluctuations. Both left and right wing lengths were measured as described previously [20]. In short, both wings were removed, dry-mounted on microscope slides, and photographed with a Leica EZ4 D dissection microscope (Leica Microsystems, Suisse). Length was then measured using the software Image J1.41.0 (Wayne Rasband, National Institutes of Health, U.S.A.) from the annular notch to the end of the radius vein, excluding fringe scales. This length raised to the cube (WL^3^) was considered an index of mosquito size [20].

### Data analysis

Data were entered into Microsoft Windows Excel 2010, checked for accuracy, then imported to R studio version 3.2.0 for data manipulation, visualization and statistical analysis (Additional files 1, 2). Fisher’s exact test, P < 0.05 was accepted for statistical significance. LT_80_ survival for treatments and concentrations were determined using a generalized linear model (GLM) approach. For all bioassays, mosquitoes were considered alive if they could stand upright and dead if they were unresponsive to stimuli following the recommendations of the WHO Pesticides Evaluation Scheme [21].

## Results

### Effect of *C. anophelis *sp. nov. IRSSSOUMB001 on highly insecticide-resistant *Anopheles coluzzii* larval survival

**Fig. 1:**
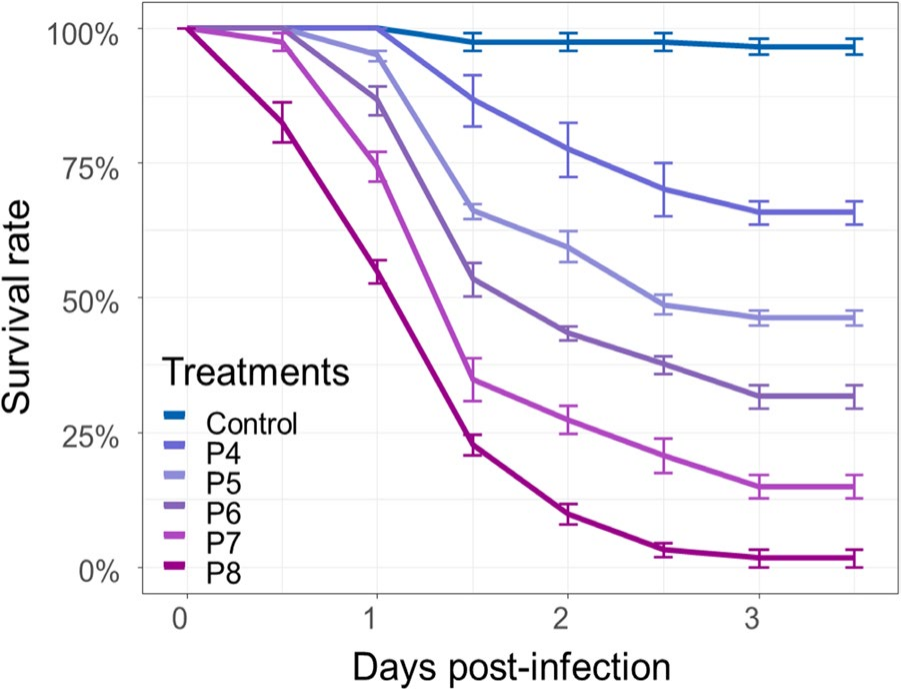
Survival curves of *An. coluzzii* L3 larvae exposed to different concentrations of *Chromobacterium anophelis* IRSSSOUMB001

We found that 96.9% of the *An. coluzzii* used for bioassays carried the *kdr* resistance gene. Within ~ 1.75 ± 0.14 days post-infection, more than 80% of mosquitoes exposed to the higher concentration to 10^8^ bacterial cells/ml were dead, significantly faster (P < 0.05) than those exposed to 10^7^ bacterial cells/ml with a LT80 of 2.62 ± 0.12 (Fig. 1, Table 2). The three lowest concentrations (10^6^,10^5^ and 10^4^ cfu/ml) did not reach the LT_80_ threshold during the observation time (Fig. 1, Table 2). Observing survival over 3 days, larvae of the uninfected control group never dropped below 93.5% survival (Fig. 1, Table 2).

**Table 2:** **LT**_**80**_ survival values of *Anopheles coluzzii* laboratory L3 larvae treated by *Chromobacterium anophelis* IRSSSOUMB001 reared under standard insectary conditions during 3 days

Treatments	LT_80_ Mean	SE
Control	–	–
P4	–	–
P5	–	–
P6	–	–
P7	2.62	0.12
P8	1.75	0.14

Pairwise comparison of LT_50_ and LT_80_ values per conidia suspension concentrations

*Control* control is exposed to any treatment, *P4* 10^4^ bacteria cells/ml, *P5* 10^5^ bacteria cells/ml, *P6* 10^6^ bacteria cells/ml, *P7* 10^7^ bacteria cells/ml, *P8* 10^8^ bacteria cells/ml, *SE* standard error of the mean

All treatments were significant at p < 0.05

### Effect *C. anophelis* sp. nov. IRSSSOUMB001 on insemination

**Fig. 2:**
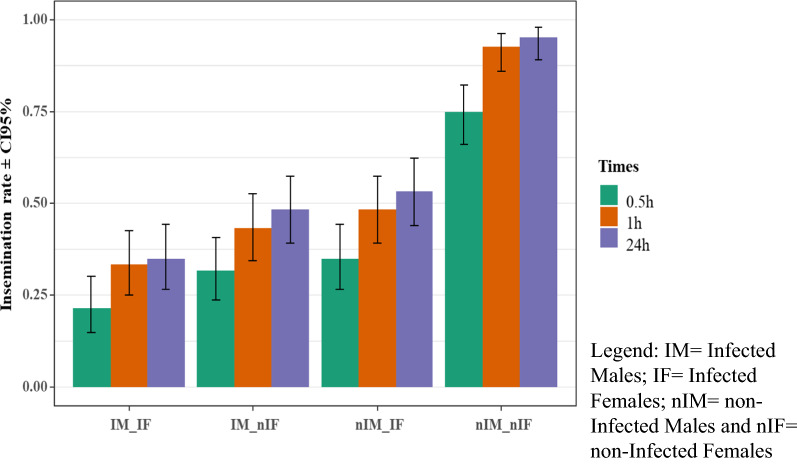
Effects of *Chromobacterium anophelis* IRSSSOUMB001 infection on insemination rates of female mosquitoes from different crossing types. *IM* infected males, *IF* Infected Females, *nIM* non-infected males, *nIF* non-infected females

The highest insemination rate was recorded in females from crosses involving uninfected males and females (Fig. 2), ranging from 95 ± 1.99% to 75 ± 3.95%). The lowest insemination rates were observed from crosses between infected males and females, ranging from 35 ± 4.35% to 21 ± 3.76%. There were no significant differences in insemination rates within different treatments (ANOVA, df = 6, P = 0.2436). Regardless of the different treatments, there were significant differences in insemination rates among the three different contact times (0.5, 1.0 and 24 h) (ANOVA, df = 3, P < 0.001)**.** The average of interactions showed statistically significant differences for most of the treatments (Table 3).

**Table 3:** Interaction average of mosquito insemination rate between different crossings based on *Chromobacterium anophelis* IRSSSOUMB001 infection

Interaction_average	P-value
IM_nIF—IM_IF	0.00876**
nIM_IF—IM_IF	< 0.001***
nIM_nIF—IM_IF	< 0.001***
nIM_IF—IM_nIF	0.60927
nIM_nIF—IM_nIF	< 0.001***
nIM_nIF—nIM_IF	< 0.001***

^**^Statistically significant

*IM*  Infected Males, *IF*  Infected Females, *nIM*  non-infected males, *nIF* non-infected females

*P-value between (0.01–0.05); **P-value less than or equal to 0.001; ***P-value less than or equal to 0.0001

### Effect of *C. anophelis* sp. nov. IRSSSOUMB001 infection on wing length of offspring from infected mother mosquitoes

**Fig. 3:**
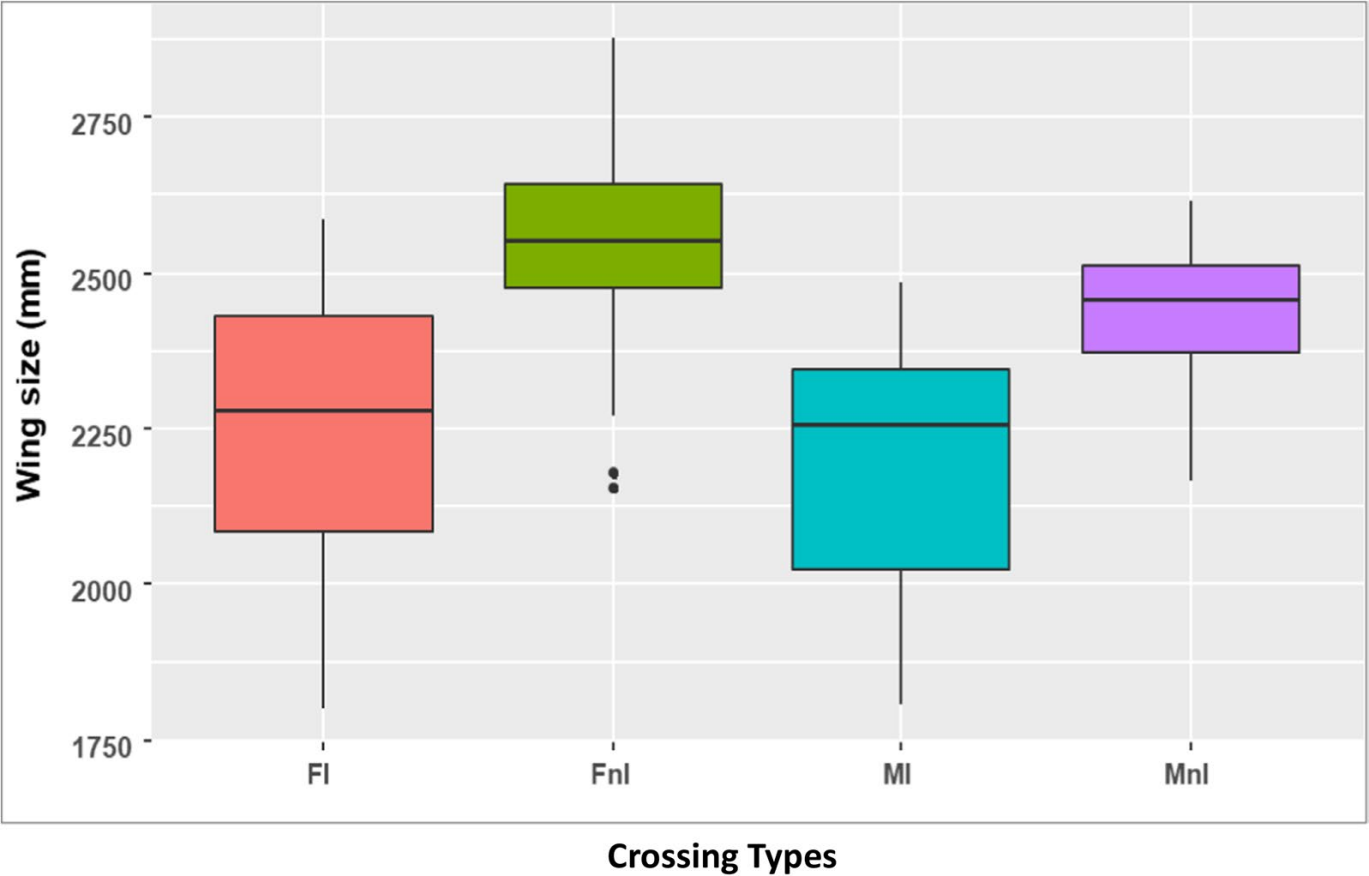
Comparison of wing size between *An. coluzzii* offsprings from *Chromobacterium anophelis* IRSSSOUMB001 infected and non-infected mothers*. IM*  infected males, *IF*  Infected Females, *nIM*  non-infected males*, nIF* non-infected females

Both female and male uninfected mosquitoes were significantly larger than infected mosquitoes as determined by the wing length assay (Fig. 3, df = 3, P < 0.001). Within females, the average of wing size was 2.1 ± 0.2 mm and 2.55 ± 0.17 mm for offspring from infected females and uninfected females respectively. For males, the average wing size was 1.99 ± 0.15 mm, and 2.44 ± 0.13 mm for offspring from infected and uninfected males respectively (Fig. 3).

## Discussion

Chromobacteria are naturally occurring soil bacteria. Some species produces a number of compounds that contribute to the formation of several complex modes of action, creating biopesticides that are highly active against agricultural pests [22]. It has been specifically shown in the current study that the exposure of L3-larvae insecticide-resistant An. coluzzii to different concentrations of *C. anophelis* sp. nov. IRSSSOUMB001 resulted in high rates of mortality. This strain has previously been shown to be highly virulent against adults [10]. The larvicidal activity of this strain of *Chormobacterium* could be the direct result of a specific mosquitocidal factor or factors, or by systemic infection and dissemination into the hemolymph. Alternatively, colonization of the midgut might cause mortality indirectly by interfering with vital functions of the mosquito [8]. Although there is as yet no definitive connection between *Chromobacterium* spp. secondary metabolites and insecticidal activity, among the potential virulence factors that may contribute to mosquitocidal activity are the production of siderophores, hydrogen cyanide, exoproteases, Histone deacetylase (HDAC) inhibitors such as romidepsin, or chitinases [8]. In addition, some strains of *Chromobacterium* are capable of forming biofilms in vitro [8], though whether biofilm formation occurs within the mosquito midgut or other organs remains untested. For future studies, formulations could be developed for introducing and disseminating *C. anophelis* IRSSSOUMB001 as both mosquito larvicides and adulticides. Interestingly, our strain could be formulated following the preparation from Caragata et al*.* [9]. They have recently developed with another mosquitocidal strain of *Chromobacterium* Csp_P an air-dried powder containing no live bacteria formulation. This formulation can be incorporated into attractive baits and fed directly to mosquito larvae [9].

Another entomopathogenic effect in these studies was the reduction of insemination rates. The insemination rates generated by crossing infected males and females were similar to those obtained by Helinski et al. in large cages with low dose irradiation [23]. Sperm production may also be negatively affected by bacterial treatments. The low insemination rates observed in this study could also be due to the reduction of energy reserves, and thus mating capacity in infected mosquitoes. Flight and hearing capabilities, pheromone or sperm production, may also be responsible for the drop in mating efficiency and for lower insemination rates [24, 25].

Body size reduction is another trait associated with mosquito mating capacity and competitiveness success [20]. Our results showed that uninfected *An. coluzzii* offspring were significantly larger than infected ones. It was shown previously that siderophores, hydrogen cyanide, and secreted chitinases could affect the fitness of adult mosquitoes [8, 10]. *Wolbachia* spp. acts in mosquitoes by manipulating the reproduction system of their host. *C. anophelis* IRSSSOUMB001 has now also been shown to be a potent parasite of mosquito reproduction in addition to its larvicidal activity. This bacterium may also prove useful as a Sterile Insect Technique tool in the future.

## Conclusion

The present study shows that *C. anophelis* IRSSSOUMB001 is highly virulent against larvae of wild-caught malaria vector *Anopheles coluzzii*, and that this bacterium has important disruptive effects on mosquito mating and competitiveness mainly by reducing female insemination rates. Surprisingly, this bacterium also shows trans-generation impacts through a reduction of offspring sizes from infected parents. From our data, *C. anophelis.* IRSSSOUMB001 is a promising tool for malaria vector control at both larval and adult stages. However, additional studies need to be completed before definitive conclusions can be drawn about the practical utility of this bacterium for malaria control. These studies will focus on development of methods for disseminating the bacterium to wild mosquitoes, its health and environmental safety, but also on the social acceptance of this bacterium as a biological control agent.

## Supplementary Information

**Additional file 1: **The R codes of data analysis.

**Additional file 2: **Row data.

## Data Availability

The supplementary R codes and data for all analyses in this article are in supplemental files and could also be available upon request to the corresponding authors.

## Abbreviations

Sp. nov: Species nova, or new species.
LT_50_: Is the median (50%) Lethal Time (time until death) after exposure of a mosquito to bacterial infections;
LT_80_: Is the 80% Lethal Time (time until death) after exposure of a mosquito bacterial infections;
IM: Infected males
IF: Infected females
nIM: Non-Infected males
nIF: Non-Infected females
HDAC: Histone deacetylase inhibitor
