# Supplementary material for: Infection of the malaria vector Anopheles coluzzii with the entomopathogenic bacteria Chromobacterium anophelis sp. nov. IRSSSOUMB001 reduces larval survival and adult reproductive potential
Source: Malar J. 2023 Apr 13;22:122. doi: 10.1186/s12936-023-04551-0 (PMC10103495; doi:10.1186/s12936-023-04551-0)
Supplement: Supplementary file 1 — Additional file 1: The R codes of data analysis. [file 12936_2023_4551_MOESM1_ESM.pdf]

Title: " **Infection of the malaria vector** *Anopheles coluzzii* **with the entomopathogenic bacteria** *Chromobacterium sp. Burkina (C.sp_B)* **highly reduces larval survival and adult reproductive potentials**"

Authors: "Etienne BILGO" and " E. Jacques GNAMBANI "

Date: "31st October 2022"

Larval Survival Analysis

Data importation

```
dat<- read.table("larves.txt",header=TRUE,stringsAsFactors = T)
View(dat) colnames(dat)

 # Data manupilations

colnames(dat)

library(reshape2)

 #melt(Data set, column names to keep, column names to restructure) colnames(dat)

df=melt(dat, colnames(dat)[c(1,9)], colnames(dat)[2:8])

 View(df)

 library(plyr)

 df2=ddply(df, .(Replicate,variable), transform, nval=sum(value, na.rm=TRUE))
View(df2) df3=ddply(df2, .(Replicate,variable), transform, Dead=cumsum(value))
View(df3) df3$Survival=(1df3$Dead/df3$nval)*100 View(df3) colnames(df3)[3]="Treat
ments"

 View(df3)

df5=subset(df3, Day!="Alives") df5$Day=as.numeric(as.character(df5$Day))

View(df5)

df6=ddply(df5, .(Day,Treatments), summarize, mean=mean(Survival),
replicates=length(Survival), se=sd(Survival)/sqrt(length(Survival)))
```

```
View(df6)
library(ggplot2)
limits=aes(ymax=mean+se, ymin=mean-se)
theme = theme_bw()+theme(text = element_text(size=25),
                axis.title.x = element_text(size=25),
                title = element_text(size=25),
                legend.title = element_text(size=25),
                legend.text = element_text(size=20))
cbPalette <-c(117,139,130,95,134,33,2,40,51,72)
Plt=ggplot(df6, aes(Day, mean,
color=Treatments))+geom_line(size=2)+geom_errorbar(limits, width=.1,
size=1)+theme+scale_colour_manual(values=cbPalette)+ylab("Survival rate
(%)")+xlab("Days")
#Displays the plot
Plt

## Calculation of LT50s ##
####Load packages####
library(tidyverse)
library(reshape2)
library(plyr)
library(scales)
library(survival)
library(MASS)
View(df5)
LTdat=df5
View(LTdat)
LTdat$Alives=LTdat$nval-LTdat$Dead
View(LTdat)
attach(LTdat)
surv.per=0.50
```

```
colnames(LTdat)
LTdat2=ddply(LTdat, .(Treatments, Replicate),
summarize,LT=as.numeric(dose.p(glm(cbind(Alives,Dead)~Day,binomial),p=surv.per)))
View(LTdat2)
LTdat2[LTdat2$LT>6|LTdat2$LT<0,]$LT=NA
LT50.Error=ddply(LTdat2, .(Treatments), summarize,"LT50 Mean"=mean(LT,na.rm=T),
se=sd(LT,na.rm=T)/sqrt(length(LT[!is.na(LT)])),Replicates=length(LT[!is.na(LT)]))
View(LT50.Error)
### Insemination Data Analysis ###
## Data importation ##
data <- read.table("Data_Ins.txt",header=TRUE,stringsAsFactors = T)
str(data)
summary(data)
table(data$Contact,data$Treatment)
attach(data)
View(data)
y <- cbind(Inseminated,Non_inseminated)  # Variable de reponse binomiale
##GLMM##
library(glmmTMB)
mod<-glmmTMB(y~Treatment*Contact+(1|Replicate), family="binomial")
summary(mod)
mod2 <- update(mod,~.-Treatment:Contact)
anova(mod,mod2, test="Chi")    ### Il n'y a pas d'effet siginificatif de l'interaction
Treatment:Contact
summary(mod2)
mod3 <- update(mod2,~.-Contact)
anova(mod2,mod3, test="Chi")   ### Il y a un effet siginificatif du temps de Contact sur
le % d'insemination
library(multcomp)
summary(glht(mod2,linfct = mcp(Contact="Tukey"),interaction_average = F))
#               Estimate Std. Error z value Pr(>|z|)
```

```
#1h - 0.5h == 0    0.6929     0.1478   4.687   <1e-04 ***
#24h - 0.5h == 0   0.8674     0.1482   5.854   <1e-04 ***
#24h - 1h == 0     0.1745     0.1434   1.217    0.443
summary(mod2)
mod4 <- update(mod2,~.-Treatment)
anova(mod2,mod4, test="Chi")   ### Il y a un effet siginificatif du traitement sur le % d'insemination
library(multcomp)
summary(glht(mod2,linfct = mcp(Treatment="Tukey"),interaction_average = F))
#                       Estimate Std. Error z value Pr(>|z|)
#MI_FnI - MI_FI == 0     0.5024     0.1595   3.150  0.00876 **
#MnI_FI - MI_FI == 0     0.6896     0.1587   4.344  < 0.001 ***
#MnI_FnI - MI_FI == 0    2.8764     0.2004  14.352  < 0.001 ***
#MnI_FI - MI_FnI == 0    0.1872     0.1531   1.223  0.60927
#MnI_FnI - MI_FnI == 0   2.3740     0.1955  12.145  < 0.001 ***
#MnI_FnI - MnI_FI == 0   2.1868     0.1946  11.239  < 0.001 ***
library(car)
Anova(mod,Type=II)
#                     Chisq Df Pr(>Chisq)
#Treatment         191.3870  3    < 2e-16 ***
#Contact            36.6516  2    1.1e-08 ***
#Treatment:Contact   7.4774  6     0.2789
#Plotting#
library(emmeans)
library(ggplot2)
### Interactions
data.graph <- summary(emmeans(mod, ~ Treatment | Contact), type="response")
data.graph
ggplot(data=data.graph, aes(x=Treatment, y=prob, fill=Contact)) +
  coord_cartesian(ylim = c(0, 1)) +
  ylab("Insemination rate ? CI95%") +
```

```
  geom_bar(stat = "identity", position=position_dodge()) +
  geom_errorbar(aes(ymin=lower.CL, ymax=upper.CL), position=position_dodge(.9), 
width=.2)+
  scale_fill_brewer(palette="Dark2")
## Effect of treatment ##
data.graph2 <- summary(emmeans(mod2, ~ Treatment),  type="response")
data.graph2
ggplot(data=data.graph2, aes(x=Treatment, y=prob,fill=Treatment)) +
  coord_cartesian(ylim = c(0, 1)) +
  ylab("Activation rate ? CI95%") +
  geom_bar(stat = "identity", position=position_dodge()) +
  geom_errorbar(aes(ymin=lower.CL, ymax=upper.CL), 
position=position_dodge(.9),width=.1)+
  scale_fill_brewer(palette="Dark2") +
  theme (legend.position ="non")
##Impact of contact time##
data.graph3 <- summary(emmeans(mod2, ~ Contact), type="response")
data.graph3
ggplot(data=data.graph3, aes(x=Contact, y=prob, fill=Contact)) +
  coord_cartesian(ylim = c(0, 1)) +
  ylab("Activation rate ? CI95%") +
  geom_bar(stat = "identity", position=position_dodge()) +
  geom_errorbar(aes(ymin=lower.CL, ymax=upper.CL),position=position_dodge(.9), 
width=.1)+
  scale_fill_brewer(palette="Dark2")+
  theme(legend.position ="non")
## Wing data analysis ###
data <- read.table("Data_wing_female.txt",header=TRUE,stringsAsFactors = T)
str(data)
summary(data)
attach(data)
```

```
hist(Wing_size,freq=FALSE, col="grey")
lines(density(Wing_size), col="red")
library(car)
qqPlot(Wing_size)
shapiro.test(Wing_size) # W = 0.55961, p-value < 2.2e-16 # No de normality
fligner.test(Wing_size,Treatment)
# X? = 8.8591, df = 3, p-value = 0.03122 # No homogeniity of variances
kruskal.test(Wing_size~Treatment)
# chi-squared = 69.446, df = 3, p-value = 5.609e-15
x <- aov (Wing_size~Treatment)
summary(x)
#             Df  Sum Sq Mean Sq F value   Pr(>F)
#Treatment     3  263878   87959   8.081 3.97e-05 ***
TukeyHSD(x)
#              diff        lwr        upr     p adj
#FnI-FI  -84.113725 -135.55882 -32.668635 0.0001983
#MI-FI   -49.549020 -103.03908   3.941038 0.0804125
#MnI-FI  -86.477567 -138.12258 -34.832553 0.0001304
#MI-FnI   34.564706  -16.88038  86.009796 0.3058252
#MnI-FnI  -2.363842  -51.88776  47.160077 0.9993237
#MnI-MI  -36.928548  -88.57356  14.716467 0.2523871
### GLM ###
mod<-glm(Wing_size~Treatment)
summary(mod)
mod2 <- update(mod,~.-Treatment)
anova(mod,mod2, test="Chi")
library(car)
Anova(mod)
#          LR Chisq Df Pr(>Chisq)
#Treatment   24.244  3  2.222e-05 ***
```

```
library (multcomp)
summary(glht(mod,linfct = mcp(Treatment="Tukey"),interaction_average = F))
#              Estimate Std. Error z value Pr(>|z|)
#FnI - FI == 0   -84.114     19.870  -4.233   <0.001 ***
#MI - FI == 0    -49.549     20.660  -2.398   0.0775 .
#MnI - FI == 0   -86.478     19.947  -4.335   <0.001 ***
#MI - FnI == 0    34.565     19.870   1.740   0.3030
#MnI - FnI == 0   -2.364     19.128  -0.124   0.9993
#MnI - MI == 0   -36.929     19.947  -1.851   0.2493
library(ggplot2)
ggplot(data, aes(x=Treatment, y=Wing_size, fill=Treatment)) +
  geom_boxplot()+
  ylab("Wing size")+
  theme(legend.position ="non")
library(Rmisc)
data.graph <- summarySE(data, measurevar="Wing_size", groupvars="Treatment")
data.graph
ggplot(data=data.graph, aes(x=Treatment, y=Wing_size, fill=Treatment)) +
  coord_cartesian(ylim = c()) +
  ylab("Wing size ? se") +
  geom_bar(stat = "identity", position=position_dodge()) +
  geom_errorbar(aes(ymin=Wing_size-se,
ymax=Wing_size+se),position=position_dodge(.9), width=.1)+
  scale_fill_brewer(palette="Dark2")+
  theme(legend.position ="non")
boxplot(Wing_size~Treatment)$out
outliers <- c(348,329,345,349)
data2<- data[-which(data$Wing_size %in% outliers),]  # retrait des outliers du jeu de
donn?e
boxplot(data2$Wing_size~data2$Treatment)
```

```
detach(data)
attach(data2)
hist(Wing_size,freq=FALSE, col="grey",  ylim=c(0,0.008))  # Normalit? OK
lines(density(Wing_size), col="red")
library(car)
qqPlot(Wing_size)
shapiro.test(Wing_size) # W = 0.99411, p-value = 0.5531 # Normalit? Ok
fligner.test(Wing_size,Treatment) # X? = 3.7306, df = 3, p-value = 0.2921
 # Homogenicity
#-----ANOVA--------#
x <- aov(Wing_size~Treatment)
summary(x)
#             Df  Sum Sq Mean Sq F value   Pr(>F)
#Treatment     3 210384   70128   36.41 <2e-16 ***
TukeyHSD(x)
#             diff        lwr        upr     p adj
#FnI-FI  -35.91492  -57.91090 -13.918937 0.0002039
#MI-FI   -49.54902  -72.05306 -27.044983 0.0000002
#MnI-FI  -86.47757 -108.20537 -64.749767 0.0000000
#MI-FnI  -13.63410  -35.63008   8.361875 0.3779589
#MnI-FnI -50.56265  -71.76380 -29.361502 0.0000000
#MnI-MI  -36.92855  -58.65635 -15.200747 0.0000999
mod<-glm(Wing_size~Treatment)
summary(mod)
mod2 <- update(mod,~.-Treatment)
anova(mod,mod2, test="Chi")
library(car)
Anova(mod)
#          LR Chisq Df Pr(>Chisq)
#Treatment   109.23  3  < 2.2e-16 ***
```

```
library(multcomp)
summary(glht(mod,linfct = mcp(Treatment="Tukey"),interaction_average = F))
#               Estimate Std. Error z value Pr(>|z|)
#FnI - FI == 0   -35.915      8.495  -4.228 0.000149 ***
#MI - FI == 0    -49.549      8.691  -5.701  < 1e-04 ***
#MnI - FI == 0   -86.478      8.391 -10.306  < 1e-04 ***
#MI - FnI == 0   -13.634      8.495  -1.605 0.375575
#MnI - FnI == 0  -50.563      8.188  -6.176  < 1e-04 ***
#MnI - MI == 0   -36.929      8.391  -4.401  < 1e-04 ***

ggplot(data2, aes(x=Treatment, y=Wing_size, fill=Treatment)) +
  geom_boxplot()+
  ylab("Wing size")+
  theme(legend.position ="non")
data.graph2 <- summarySE(data2, measurevar="Wing_size", groupvars="Treatment")
data.graph2
ggplot(data=data.graph2, aes(x=Treatment, y=Wing_size, fill=Treatment)) +
  coord_cartesian(ylim = c()) +
  ylab("Wing size ? se") +
  geom_bar(stat = "identity", position=position_dodge()) +
  geom_errorbar(aes(ymin=Wing_size-se,
ymax=Wing_size+se),position=position_dodge(.9), width=.1)+
  scale_fill_brewer(palette="Dark2")+
  theme(legend.position ="non")
```
